# Novel oral anticoagulants: the translation of ‘de Leidraad’ into daily clinical practice

**DOI:** 10.1007/s12471-014-0557-5

**Published:** 2014-04-23

**Authors:** J. Jaspers Focks, M. A. Brouwer

**Affiliations:** Department of Cardiology, Radboud University Medical Centre, Geert Grooteplein Zuid 10, 6525 GA Nijmegen, the Netherlands

The novel oral anticoagulants (NOACs) have been approved for stroke prevention in atrial fibrillation (AF) and are fully reimbursed by the Dutch health insurance agencies: as of December 2012 for dabigatran and rivaroxaban followed by apixaban in June 2013. To ensure optimal efficacy and safety in daily clinical practice, the Dutch Ministry of Health published a document (‘de Leidraad’) which contains a series of precautionary measures that should be addressed before initiation of the prescription of this new class of drugs [1].

The most important requirement is the presence of a hospital protocol that describes how NOAC-related bleeding complications are dealt with and how anticoagulation with these agents is managed peri-operatively. Moreover, ‘de Leidraad’ discusses the organisation of logistic issues related to the implementation of the NOACs in clinical practice, such as switching from vitamin-K antagonists to a NOAC, monitoring of renal function, and the registration of complications.

In this issue, Folkeringa and colleagues describe the implementation of ‘de Leidraad’ in the Medical Centre Leeuwarden and present the data of their first experience with NOACs [2]. To facilitate the cardiologist, the function of a specialised anticoagulation nurse was instituted as a key player in the initiation and follow-up of outpatient NOAC therapy.

For the management of a patient with AF, the effects of nurse-led care on guideline adherence seem promising when compared with standard of care, and the results of the ongoing RACE-4 study (NCT01740037) will increase the evidence on this topic and its potential impact on clinical outcome [3].

Moreover, the authors describe other potential values of the specialised nurse, for example in the central registration of patients receiving a NOAC and in maintaining the chain of care for anticoagulation therapy, a task that was formerly performed by the Thrombosis Service. The authors nicely demonstrate that about a quarter of patients contact the outpatient clinic with a variety of questions. A nurse can perform a triage, and substantially reduce the physicians’ time spent with these questions.

While the title and introduction suggest that the report on the Leeuwarden model will give insight into the implementation of ‘de Leidraad’ into daily clinical practice, the authors mainly focus on describing their population, the initiation of treatment and the first (limited) data during follow-up. It would have been interesting to know more about how the abovementioned requirements from ‘de Leidraad’ were incorporated in the ‘Leeuwarden Model’.

With regard to the management of bleeding, two issues should be addressed. With regard to logistics, there should be a 24/7 coverage to contact a physician in case of NOAC-related bleeding questions. Of note, this should be the case for bleeding on vitamin-K antagonists as well, as stated in the Dutch National Standard Chain of Care on Antithrombotics (Landelijke Standaard Ketenzorg Antistolling). In fact, the introduction of NOACs has resulted in renewed awareness of aspects that were already part of the ‘chain of anticoagulation care’. A second aspect concerns the availability of a hospital protocol on bleeding complications that also incorporates NOAC-related bleeding.

In the Leeuwarden registration, peri-operative advice comprises only a minority of the questions so far. It should be emphasised, however, that multidisciplinary awareness of NOACs is of the utmost importance. In addition to a key player, a so-called ‘case manager’, the regional availability of a (multidisciplinary) protocol, which also addresses peri-operative issues, could improve overall care (e.g. www.necf.nl). Of note, it is important to ensure that dentists and general practitioners (GPs) are aware of this protocol.

Even more important than with the start of any other drug, a letter to the GP is a key element of patient care. As GPs will not prescribe NOACs themselves, they are not as familiar with these drugs as with other drugs. Although dose adjustments of the NOAC can only be performed by the prescriber, it is important to inform the GP when dose adjustment is indicated, to make sure that the patient is referred on time if, for example, renal function deteriorates.

Many randomised trials have demonstrated that NOACs are a safe and efficacious alternative to vitamin-K antagonists in patients with AF. Appreciation of the abovementioned aspects could further enhance their efficacy and safety in daily clinical practice.

It should be noted that during the various trials there was no or minimal experience with these agents and clear protocols on how to act in case of complications were mostly absent. Nonetheless, all NOACs proved to be at least as safe as warfarin. Furthermore, a post-hoc analysis studying the management and outcomes of patients with major bleeding complications demonstrated that patients who received dabigatran had a shorter stay at the intensive care unit, with a trend towards lower mortality when compared with warfarin therapy [4].

Finally, the authors report that negative publicity has also been a reason for patients not to use a NOAC. Statements concerning the absence of an antidote contribute to this, as well as reports on an allegedly marked risk of gastrointestinal bleeding. When informing the patient, it should be realised that the management of major bleeding does not differ between NOACs and, for example, acenocoumarol. For bleeding complications with the latter, vitamin K can only be used as an antidote in non-urgent situations, given its slow mode of action. Reports on a markedly increased risk of gastrointestinal bleeding should be interpreted in the context that many of the patients studied in these analyses used NOACs for indications that are not endorsed in daily clinical practice [5].

As for patients with AF, the risk of gastrointestinal bleeding is only slightly increased for dabigatran 150 mg twice daily and rivaroxaban, and should be put in perspective to the interesting overall safety profile (lower intracranial haemorrhage). For the other NOACs no increased risk of gastrointestinal bleeding is observed [5]. In analogy to the use of other antithrombotic agents, the guidelines with regard to the prescription of proton pump inhibitors should be followed.

In conclusion, Folkeringa and colleagues should be commended for the emphasis they put on the ‘chain of care’ and a nurse-coordinated outpatient anticoagulation clinic could be an attractive option to facilitate the physician. Before local implementation of a similar strategy, we refer to the valuable aspects mentioned in ‘de Leidraad’ to ensure that this promising new class of drugs will reach its full potential in daily clinical practice as well.
